# Methacrylated gelatin/hyaluronan-based hydrogels for soft tissue engineering

**DOI:** 10.1177/2041731417744157

**Published:** 2017-12-21

**Authors:** Lukas Kessler, Sandra Gehrke, Marc Winnefeld, Birgit Huber, Eva Hoch, Torsten Walter, Ralf Wyrwa, Matthias Schnabelrauch, Malte Schmidt, Maximilian Kückelhaus, Marcus Lehnhardt, Tobias Hirsch, Frank Jacobsen

**Affiliations:** 1 Department of Plastic Surgery and Burn Centre, BG University Hospital Bergmannsheil GmbH, Ruhr University Bochum, Bochum, Germany; 2 Research and Development, Beiersdorf AG, Hamburg, Germany; 3 Institute for Interfacial Engineering and Plasma Technology, University of Stuttgart, Stuttgart, Germany; 4 Biomaterials Department, INNOVENT e. V., Jena, Germany

**Keywords:** Soft tissue defects, adipose-derived stem cells, adipose tissue, methacrylation

## Abstract

In vitro–generated soft tissue could provide alternate therapies for soft tissue defects. The aim of this study was to evaluate methacrylated gelatin/hyaluronan as scaffolds for soft tissue engineering and their interaction with human adipose–derived stem cells (hASCs). ASCs were incorporated into methacrylated gelatin/hyaluronan hydrogels. The gels were photocrosslinked with a lithium phenyl-2,4,6-trimethylbenzoylphosphinate photoinitiator and analyzed for cell viability and adipogenic differentiation of ASCs over a period of 30 days. Additionally, an angiogenesis assay was performed to assess their angiogenic potential. After 24 h, ASCs showed increased viability on composite hydrogels. These results were consistent over 21 days of culture. By induction of adipogenic differentiation, the mature adipocytes were observed after 7 days of culture, their number significantly increased until day 28 as well as expression of fatty acid binding protein 4 and adiponectin. Our scaffolds are promising as building blocks for adipose tissue engineering and allowed long viability, proliferation, and differentiation of ASCs.

## Introduction

Tissue defects due to trauma, tumor removal, or diseases are mainly treated with autologous tissue transfer or allogenic tissue. However, there are limited autologous sources for these treatments, and they come at the expense of donor site morbidity or immunogenic reactions of the allogenic transplant. An artificial vascularized soft tissue substitute could offer alternative strategies. Various approaches have been made to create tissue-engineered adipose tissue, and quite a few have been shown suitable for this purpose. The choice of the scaffold material is of utterly importance in tissue engineering, and therefore, researchers have made many advances in the field of biomaterials. Basically, there are synthetic and naturally derived scaffold biomaterials, and they hold different advantages. Some examples of common synthetic biomaterials in adipose tissue engineering are silicones, poly-(ethylene) glycol, and poly-(lactic acid).^1–3^ The advantage of these materials is that their production is controllable in terms of specific material and degradation properties; furthermore, some of these materials support adipogenesis in vitro and in vivo.^1^ Likewise, scaffolds made of components generated by biologic systems like collagen, hyaluronan, and fibrinogen show good biocompatibility and support adipogenesis; but there is limited ability to control their production, and as a result, some are not well utilized yet. Nonetheless, naturally derived materials are better absorbed by the human body and cause less immune reaction when compared with synthetic ones. Hyaluronic acid (HA) is a naturally occurring polysaccharide which is ubiquitous in the human body and constitutes an important part of the extracellular matrix (ECM). Among one of its various biological functions, HA has been identified as a key player in human embryonic development. Additionally, it induces increased adipogenesis in vitro and in vivo tissue engineering^4–7^ and plays a role in angiogenesis and wound healing.^8^ Gelatin (G) is another naturally derived material consisting of partially degraded collagen—by virtue of being partially degraded it presents with less antigenicity when compared with collagen. G can be used to create a vast number of shapes with little effort. It provides structural stability and contains cell adhesion–enhancing amino acid sequences. It has been shown to increase adipogenesis in vivo.^9,10^ Both HA and G have traits which seem perfect for soft tissue engineering. Hydrogels are gels with unique characteristics. They contain hydrophilic polymers, which can form porous structures and absorb high amounts of water.^11,12^ Hydrogels are already used in the clinic, which makes them appealing for biomedical research and tissue engineering.^13,14^

In addition to materials, cells are another key player in adipose tissue engineering. Adipose-derived stem cells (ASCs) have emerged in the spotlight over the past few years and gained in importance for tissue engineering purposes^15,16^ because of their fibroblast-like morphology and multipotency, that is, ability to differentiate into all mesenchymal cell types and various other cell types.^17–19^ Approaches using other heterogeneous human cell populations or mature adipocytes or murine 3T3-L1 preadipocytes have been investigated as well,^20–23^ which all showed cytocompatibility of their constructs and differentiation of the various cell types. Despite their similarity to bone-marrow stem cells (BMSC), ASCs express different cell surface markers.^24^ However, there is no consistent consensus on the surface marker of ASCs and no highly specific marker set-up so far, which makes it difficult to characterize them properly.^25^ Many studies have been done using certain scaffold materials to culture ASCs, but only a few have looked into gelatin and hyaluronan composite hydrogels. Those studies mainly focused only on the material structure, biocompatibility, or cellular interactions for short time periods but nonetheless proved their beneficial properties for tissue engineering and bioprinting and characterized their mechanical properties extensively.^6,7,26–28^ Despite that sufficient adipose tissue construct have not been proposed so far, and therefore, we combined methacrylated gelatin and methacrylated hyaluronan (HyaMA) to generate three-dimensional (3D) scaffolds in which ASCs could form adipose tissue. The aim of this study was to imbed previous findings and combine them to create sufficient tissue constructs. For this, we focused on the analysis of the viability, proliferation, differentiation, and angiogenic potential of ASCs in the scaffold over longer periods of time.

## Methods and materials

### Isolation of ASCs and cell culture

HASCs were harvested from patients undergoing elective reconstructive surgery, for example, liposuction, breast reconstruction (deep inferior epigastric perforators (DIEP) flap), or abdominoplasty. Patients were mainly women (>82%) with a mean age of 46.7 years and signed an informed consent. Adipose tissues from surgery were transported and prepared under sterile conditions. The cells were isolated according to an established protocol and cultivated up to passage 5 in the following way. Pads of tissue were manually minced using sterile scalpel and scissors, and residual skin was removed. Minced tissue was washed a few times with sterile Dulbecco’s phosphate–buffered saline (DPBS) (PAN Biotech, Aidenbach, Germany) and then digested with collagenase I (Worthington, Lakewood, USA) in a 1:2.5 fat to collagenase ratio on a shaker for 1 h at 37°C at 220 r/min. Digested tissue was filtered through a 100-µm cell strainer (BD Bioscience, Heidelberg, Germany) and centrifuged at 1500 r/min for 5 min. Supernatant was discarded, and pellet was resuspended in red blood cell lysis buffer and incubated on ice for 8 min. Cold medium was added and filtered through a 70-µm cell strainer (BD Bioscience) and centrifuged at 1400 r/min for 5 min. The pellet was suspended in standard culture medium consisting of 1:1 Dulbecco’s modified Eagle’s medium (DMEM) (PAN Biotech), Ham’s F12 (PAN Biotech) and 10% fetal calf serum (FCS) (GE Healthcare Life Sciences, South Logan, USA), and 1% penicillin/streptomycin (PAN Biotech). Cells were counted using CASY-ONE cell counter (Omni Life Sciences, Bremen, Germany) and then seeded in cell culture flasks (Sarstedt, Nümbrecht, Germany). Medium was changed every 2–3 days. Cells were cultivated till they reached 70%–90% confluence and then passaged or used for experiments. For the in vitro angiogenesis assay, human umbilical vein endothelial cells (HUVECs) were purchased and cultured with endothelial growth medium endothelial cell growth medium 2 (EGM-2) (Lonza, Cologne, Germany). HUVECs were cultured no further than passage 5 for experiments.

### Adipogenic differentiation

Differentiation of ASCs to adipocytes was induced by an established protocol.^21^ A volume of 0.5 mM of 3-isobutyl-1-methylxanthin (AppliChem, Darmstadt, Germany), 200 µM of indomethacin (Biotrend, Cologne, Germany), 1 µM of dexamethasone (Tocris Bioscience, Bristol, UK), and 10 µg/mL of insulin (Sigma-Aldrich, Taufkirchen, Germany) were added to standard culture medium. Adipogenic differentiation was evaluated by Oil-Red O or AdipoRed staining after 3–4 weeks.

### Photoinitiators

Lithium phenyl-2,4,6-trimethylbenzoylphosphinate (LAP) is a water-soluble photoinitiator (PI). LAP was synthesized as previously described.^29^ The PI was used to polymerize GelMA and HyaMA solutions to hydrogels with 365-nm light. Eosin and LAP PIs were used in concentrations as previously described.^21,30^

### Cytotoxicity of PIs

Potential cytotoxic effects of the PIs were analyzed with MTT cytotoxicity assay (Sigma-Aldrich). The yellow tetrazolium dye MTT (3-(4,5-dimethylthiazol-2-yl)-2,5-diphenyltetrazolium bromide) is reduced to purple formazan by intracellular enzymes of viable cells, and the absorbance of the solution can be measured by spectrophotometry. For the cytotox assay, 3T3-L1 murine preadipocytes were cultured on a 96-well plate for 24 h with DMEM + 10% FCS + 1% penicillin/streptomycin at 37°C. The next day, the corresponding concentrations of Eosin Y (2 mg/mL) and LAP (0.5 µg/µL) used for hydrogel cross-linking were added to the culture and incubated for 24 h/48 h. MTT was added according to the manufacturers’ protocol and was incubated with cells for 4 h at 37°C protected from light. Subsequently, the dye solution was removed and cells were lysed with dimethyl sulphoxide (DMSO) (Carl Roth, Karlsruhe, Germany), and glycine buffer was added prior to spectrophotometry reading.

### Synthesis of methacrylated gelatin and hyaluronan

Methacrylated gelatin (GelMA) was synthesized according to a procedure previously described by Hoch et al.^31^ and Van Den Bulcke et al.^32^ HyaMA was prepared as described by Moller et al.^33^ Methacrylated gelatin and hyaluronan was obtained by treatment with glycidyl methacrylate (10-fold excess). The reaction was stopped by adding glycine to the reaction mixture. Subsequent dialysis and lyophilization led to methacrylated hydrogel components.

### Hydrogel preparation

Two stock solutions of lyophilized methacrylated gelatin were made by solving gelatin in sterile phosphate buffer saline (PBS) to 10% and 20% GelMA stock solutions. A 2% HyaMA stock solution was prepared in the same way. The stock solutions were kept at 4°C in dark place for a maximum of 1 week. For the hydrogel fabrication, the GelMA and HyaMA stocks were warmed to 37°C and 6 µL 0.795 g/mL triethylamin (TEA) (Merck, Darmstadt, Germany) in sterile H_2_O with pH 7.2, and medium or cell suspension was added and intermixed corresponding to the calculated concentrations and volumes. The cross-linker LAP was added to the mix, and cloning cylinders were filled with 100 µL of the solution. Cross-linking was performed for 5 min under a ultraviolet (UV) light with 365 nm wavelength. GelMA hydrogels of 5% and 10% and a composite hydrogel of 4% GelMA/1% HyaMA (composite) were fabricated with 1 × 10^6^ incorporated ASC/mL gel solution. Additionally, we modified the composite mixture for the angiogenesis assay by adding 1.6-mg fibrinogen (Sigma-Aldrich) and 0.02 units thrombin (Sigma-Aldrich) per mL gel suspension. Due to its four main components, the mixture was called quattroGel.^34^

### Swelling

Hydrogels were weighed immediately after hydrogel gelation (*Ws*) and placed in a vacuum dryer (Savant Speed Vac). They were dried at 45°C with negative pressure for 24 h. The next day, dried gels were weighed again (*Wd*), and swelling ratio (*Q*) was calculated as previously described^35–37^

$$Q=\frac{Ws}{Wd}$$

Dried hydrogels were placed in distilled water and rehydrated. They were removed from the water at defined time points, excess water was removed, and they were weighed again.

### Viability assay

ASCs were seeded on prepared hydrogels (*n* = 9) in 96-well plates and incubated with AlamarBlue^®^ cell viability assay reagent (Lifetechnologies, Carlsbad, USA) for 24 h at 37°C protected from light. AlamarBlue^®^ reagent is a water-soluble, non-toxic dye that is converted by cells with metabolic activity. The colorimetric change or a fluorescent signal can be measured by fluorometry or spectrophotometry at 562–630 nm. Corresponding hydrogels (*n* = 3) without cells were used to normalize the absorbance of each group. Viability was measured after 1, 7, 14, and 21 days of culture with Elx 808 microplate reader (BioTek Instruments, Inc., Bad Friedrichshall, Germany), and ASCs seeded on cell culture polystyrene were used as controls. The cell viability was calculated over time. Furthermore, the viability of ASCs on different gels with standard medium (SM) and adipogenic differentiation medium (ADM) was measured.

### Microscopy

#### Live/dead staining of hydrogels

For evaluation of live/dead cell ratio, the hydrogels were cut longitudinally with a scalpel and placed in wells. The halves were carefully washed once with PBS, and the cytoplasma of living cells stained with 5 µg/mL Calcein AM (BD Bioscience) and the free DNA of dead cells with 5 µg/mL propidium iodide (Sigma-Aldrich) for 30 min. Then, staining solution was discarded and PBS added.

#### Triglyceride/nuclei/cytoplasm staining

Accumulated triglycerides or fatty acids were stained with the fluorescent dye AdipoRed (Lonza). Additionally, nuclei were stained with Hoechst 33342, and cytoplasm of vital cells was stained with fluoresceindiacetate (FDA). Hydrogels were cut and washed as described before and then incubated with 1 µg/mL Hoechst 33342 at room temperature in a dark place for 15 min. After incubation, AdipoRed^TM^ was added according to the manufacturers’ protocol, and FDA of 100 µg/mL was added to the mix. The gels were again incubated in a dark place for 15 min at room temperature. Finally, staining solution was discarded, and the gel washed twice.

The stained hydrogels were placed on microscopy slides and examined with an inverse Olympus X83 microscope (Olympus, Hamburg, Germany).

### Histology

Whole constructs were fixed in 4% paraformaldehyde in PBS overnight at 4°C and embedded in optimized cooling temperature medium (Tissue-Tek O.C.T Compound) and frozen in the vapor phase of liquid nitrogen. Cryosections of 10–15 µm were made at −21°C and stained with hematoxylin/eosin.

### Image processing and analysis

Microscopic images were taken with an inverse Olympus X83 microscope (Olympus) and the cellSens Software. After exported images of hydrogels were processed using ImageJ (National Institute of Health, Bethesda, USA), to address noise and background in fluorescent images of hydrogels, the background was subtracted and brightness/contrast was fitted.

### In vitro angiogenesis

In vitro angiogenesis was analyzed with a tube formation assay. The assay was performed according to manufacturers’ protocol (ibidi, Martinsried, Germany).^38^ Matrigel (BD Bioscience) was used as a positive control. Hydrogels were placed in triplicates in µ-slides (ibidi, Martinsried, Germany), and HUVECs or HUVEC spheroids were seeded on them and cultured with endothelial growth medium EGM-2 (Lonza) for 20 h. Phase-contrast and fluorescent images of the Calcein AM (BD Biosciences)–stained tubes and sprouts were taken and processed using the Angiogenesis Analyzer plugin for ImageJ (National Institute of Health).

### RNA isolation

Total RNA was isolated on Days 0 (control, before differentiation) and 7 using the RNeasy Lipid Tissue Mini Kit (Qiagen, Hilden, Germany) according to the manufacturers’ instructions. Before starting RNA isolation, a stainless steel bead (5 mm, Qiagen) was added to a 2-mL tube (containing 1 mL Qiazol), and gels were disrupted for 2 min using a handheld rotor-stator homogenizer (TissueRuptor, Retsch, Germany). RNA was quantified using the NanoDrop ND-1000 instrument (Peqlab Biotechnologies, Wilmington, USA). We used the Experion RNA Analysis Kit (BioRad, Munich, Germany) to check the RNA quality. The Experion-automated electrophoresis system employs LabChip microfluidic technology to automate electrophoresis for RNA analysis and offers determination of total RNA and messenger RNA (mRNA) purity and concentration at nanogram levels. The RNA integrity number (RIN) is a numerical assessment of the integrity of RNA. A RIN over 8 indicates a high quality of RNA. In our test, we observed RIN numbers of 9.7–9.8.

### Complementary DNA

Subsequently, RNA was transcribed into complementary DNA (cDNA) using the high-capacity cDNA Reverse Transcription Mix (Applied Biosystems, Life Technologies, Ltd, Paisley, UK) according to the manufacturers’ instructions.

### Low-density assay

Gene expression levels of 48 genes associated with fat metabolism were determined using *low-density assay* (LDA, for according gene list, see Supplementary File). This experiment was carried out with hASCs obtained from three different donors. All cycle threshold (CT) values were normalized to 18 s, and the expression was normalized to undifferentiated cells (day 0, control). Expression levels were calculated using the 2^–ΔΔCT^ method.

### Statistics

Data are reported as mean ± standard error of the mean (SEM), and all statistical analyses were performed with GraphPad Prism 6.0.1. Moreover, data were analyzed with student *t*-test or analysis of variance (ANOVA) if more than two groups were compared. Differences were considered as statistically significant with a *p* value <0.05.

## Results

Methacrylated gelatin and composite methacrylated gelatin and hyaluronan hydrogels of 5% and 10% were fabricated with encapsulated hASCs.

### Characterization of hydrogels and cells

**Figure 1.**
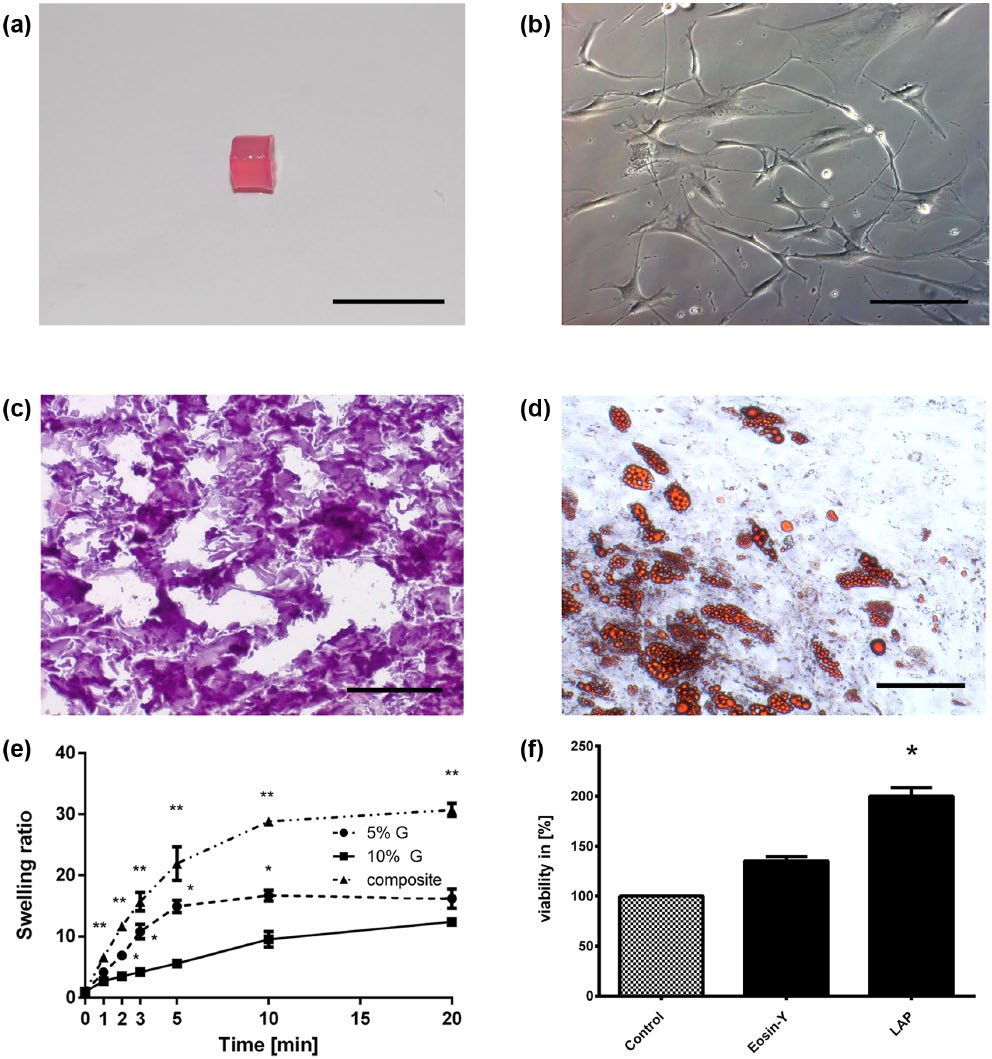
Characterization of hydrogels and adipose-derived stem cells: (a) macroscopic image of a gelatin/hyaluronic acid hydrogel with a discoid/cylindrical shape, scale: 1 cm, (b) hematoxylin/eosin staining of a hydrogel cryosection showing a porous structure, scale: 100 µm, (c) isolated adipose-derived stem cells have fibroblast-like morphology after cultivation in a culture flask, scale: 200 µm, (d) stimulation of ASCs with adipogenic differentiation medium induces triglyceride accumulation which can be visualized with Oil-Red O staining, scale: 250 µm. Hydrogels were cross-linked and weighed after gelation (*Ws*). Gels were then vacuum dried for 24 h and weighed again (*Wd*). (e) Swelling ratio (*Q*) was calculated as Q=Ws/Wd, and there was significantly higher (*p* < 0.05) swelling ratio in composite hydrogels than in methacrylated gelatin gels and (f) MTT cytotoxicity assay of LAP and eosin photoinitiators on 3T3-L1 murine preadipocytes was performed. Both photoinitiators were added to the cells in the concentration needed for successful cross-linking of hydrogels. Both LAP and eosin showed no cytotoxicity on 3T3-L1 cells. Furthermore, LAP photoinitiator increased viability of cells (*p* < 0.05). Pos. control = DMEM + 10% FCS + 1% P/S, eosin = DMEM + 10% FCS + 1% P/S + 2 mg/mL Eosin Y, LAP = DMEM + 10% FCS + 1% P/S + 0.5 µg/mL LAP.

The hydrogels were produced in a cylindrical shape (Figure 1(a)) with a porous microstructure Figure 1(c)). A porous microstructure is important for diffusion and nutrient supply within the hydrogels; therefore, we confirmed porosity by analyzing frozen sections of our hydrogels through hematoxylin and eosin staining (Figure 1(c)).

ASCs derived from adipose tissue showed fibroblast-like morphology under standard culture conditions in tissue plastic (Figure 1(b)). Since the hASCs are still an inhomogeneous cell population, we confirmed the adipogenic potential of the cells through stimulation by ADM. HASCs accumulated fatty acid in fat vacuoles within the cells in 3 weeks (Figure 1(d)) and thus showed adipogenic potential.

### Hydrogel swelling

We calculated the swelling ratios of the different hydrogels by measuring the masses of vacuum-dried and swollen hydrogel (Figure 1(e)). The swelling ratios indicate water uptake and thus can reflect uptake of nutrient-containing culture medium, which is necessary for viability, proliferation, and differentiation of encapsulated cells.^39^ Methacrylated gelatin gels showed no concentration-dependent significant differences in swelling ratios at the endpoint (Figure 1(e)). Nonetheless, 5% gelatin hydrogels did show faster swelling during the first 5 min (Figure 1(e)) (*p* < 0.05). The composite of GelMA and HyaMA had a significantly higher swelling ratio than gelatin hydrogels (*p* < 0.05), with almost 30-fold increase in weight due to water uptake, whereas gelatin hydrogels only showed a 12- to 16-fold increase in weight by swelling. All hydrogel combinations reached swelling equilibrium after about 20 min at room temperature.

### Cytotoxicity

The choice of the PI for cross-linkable hydrogels is important to ensure successful polymerization of hydrogel but should not affect the viability of the cells. Therefore, we tested the cytotoxicity of LAP and Eosin Y by MTT cytotoxicity assay (Figure 1(f)). Viability of 3T3-L1 cells was measured after 24 h of incubation with the amount of PI necessary for gelation, hence Eosin Y 2 mg/mL and LAP 0.5 µg/µL. Data indicate that neither Eosin Y nor LAP have a negative effect on the viability of cells at the employed concentration. Furthermore, viability increased significantly in cells treated with 0.5 µg/µL LAP (*p* < 0.05) when compared with an untreated control.

### Viability

**Figure 2.**
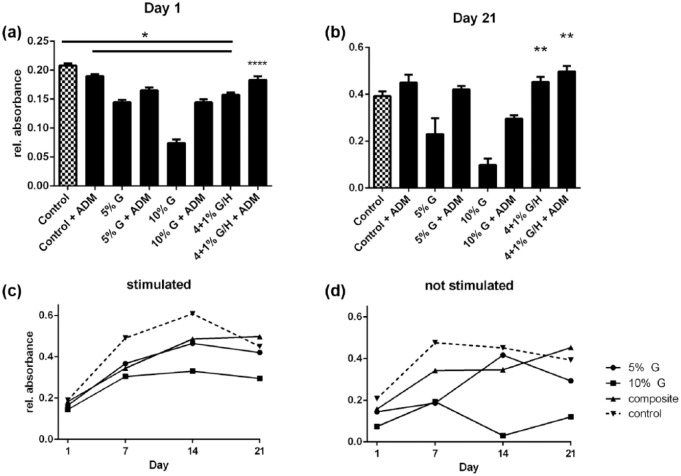
Viability of hASCs on hydrogels: (a) hASCs were transferred to hydrogel or cell culture plastic, and AlamarBlue assay was performed after 24 h. All hydrogel combinations have significantly lower cell viability (*p* < 0.05) when compared with the controls. Nonetheless, cultivation with adipogenic differentiation medium (ADM) significantly (*p* < 0.05) increased viability in each of the hydrogel groups, but not in the control group, (b) after incubating for 21 days, the viability of the cells changed. Cells cultivated with ADM still showed increased viability. ASCs cultured on gelatin/hyaluronic acid composite hydrogel showed even increased viability when compared with controls (*p* < 0.05). (c) Viability of hASC on hydrogels over 21 days under adipogenic-stimulated and (d) unstimulated conditions. Gelatin gels of 10% gelatin gels show decreased viability over time and at the endpoint. Under stimulated conditions, composite and 5% hydrogel have no significant differences in viability as observed under unstimulated conditions.

**Figure 3.**
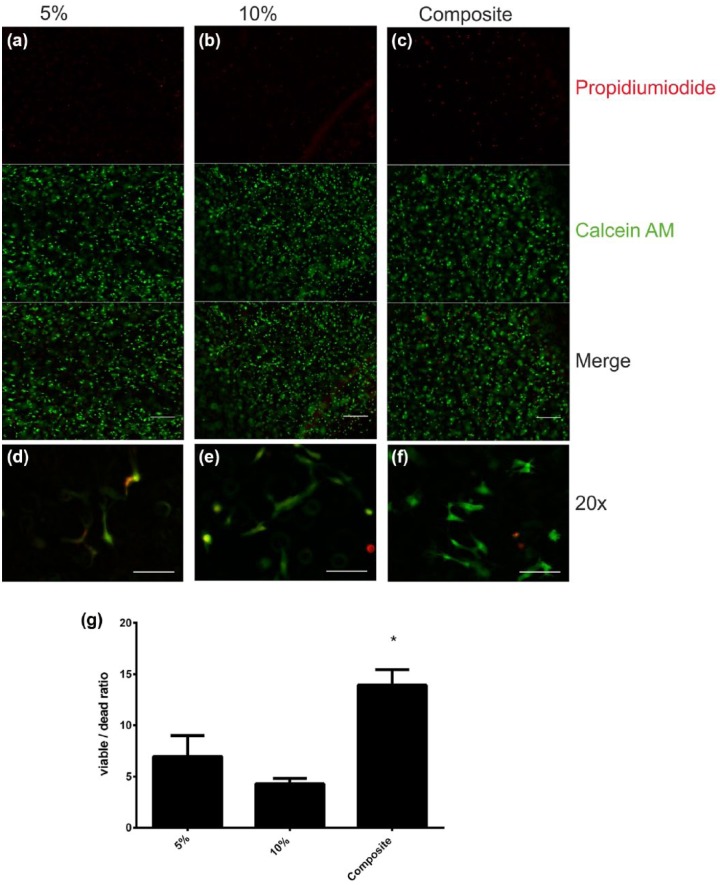
Cell viability in hydrogels: hydrogels were cultured under standard conditions for 7 days and then stained with Calcein AM/propidium iodide. Viable cells could be detected in all groups. HASCs were cultured in (a and d) 5% and (b and e) 10% methacrylated gelatin and (c and f) a composite methacrylated gelatin–hyaluronan hydrogels. Only a few dead cells could be found within the gel. Microscopically, the number of viable and dead cells appeared similar in all groups regardless the hydrogel concentration. Images were taken in 4× and 20× magnification. Scale (a–c) = 300 µm, scale (d–f) = 100 µm. (g) Significant difference in viable/dead cell ratio in composite hydrogels.

HASCs were seeded on various hydrogels and on polystyrene cell culture plastic as a control. Methacrylated gelatin hydrogels and composite hydrogels of 5% and 10% were fabricated, and ASCs were seeded on top, and either cultured with standard culture medium or ADM and viability was assessed (Figure 2). The viability of cells was calculated over time. On day 1 the control groups showed significantly higher viability than the experiment groups with the exception of the composite hydrogel cultured with ADM (Figure 2(a)). After 21 days of cultivation (Figure 2(b)), only the composite hydrogels supported similar viability to culture plastic. Additionally, ASCs cultured with ADM on composite hydrogels were significantly more viable when compared with control (*p* < 0.05). Viability over 21 days was for the stimulated ASCs when compared with unstimulated ASCs, with the exception of those cultured on the 10% GelMA, which had almost no viability (Figure 2(c) and (d)). Microscopic analysis of the hydrogel constructs with Calcein AM/propidium iodide (Figure 3) did show good cell survival after 7 days, with a significantly higher ratio of viable/dead cells for composite gels (13.93 ± 1.5) compared to 5% GelMA (6.96 ± 2.03) and 10% GelMA (4.29 ± 0.51) (*p* < 0.05) (Figure 3(g)).

### Adipogenic differentiation

#### Gene expression

**Figure 4.**
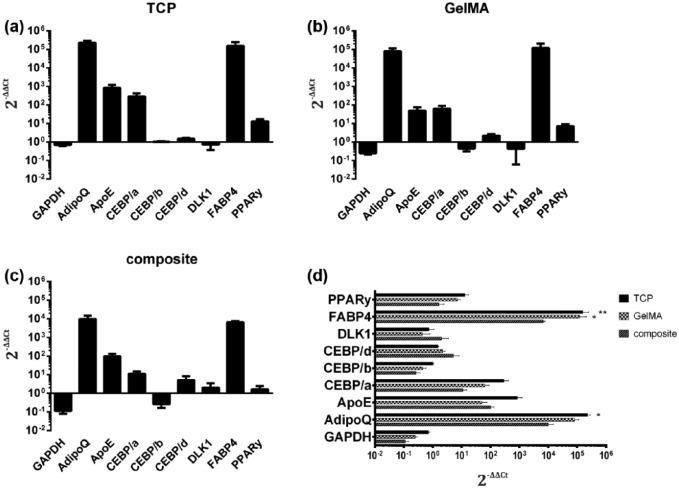
Gene expression: gene expression ratios (2^–ΔΔCT^ method) of adipocyte-specific genes of hASC were analyzed. HASCs (*n* = 3) were cultured two- and three-dimensional on: (a) TCP, (b) GelMA, and (c) GelMa/HyaMa composite gel and differentiated for 7 days. Grouped data shown in Figure 4(d) showed significant increased expression of FABP4 on TCP and GelMA compared to GelMA/HyaMA and ADIPOQ compared to the hydrogels (*p* < 0.05). The Ct values were normalized to 18 s, and additionally the expression on day 7 was normalized to undifferentiated cells on day 0.

To focus on the material’s effects on cell behavior, we first tested different hydrogel components such as GelMA and GelMA/HyaMA with regard to their effects on differentiation and gene expression changes associated with fat metabolism. At first glance, cells cultured on hydrogels showed similar expression profile of the selected genes when compared with cells cultured in two-dimensional (2D) tissue culture polystyrene (TCP) (Figure 4(a)–(c)), indicating that under all three conditions, the cells increased their fat metabolism due to differentiation induction media. Nevertheless, ASCs matured on GelMA showed a more similar gene expression profile to the 2D culture when compared with differentiated adipocytes in composite gels (GelMA/HyaMA) (Figure 4(d)), which had significantly lower levels of FABP4 compared to 2D culture and GelMa conditions and adiponectin (ADIPOQ) compared to 2D culture conditions (*p* < 0.05). The other genes of the fat metabolism were equally expressed in all groups.

In summary, preadipocytes can be differentiated in GelMA and GelMA/HyaMA and become mature adipocytes that express adipocyte-specific genes. In fact, GelMA/HyaMA mixtures induced greater alterations of gene expression compared to the 2D cell culturing; but all important genes were expressed, and there was no impact on the phenotypic differentiation during adipogenesis.

#### Microscopy

**Figure 5.**
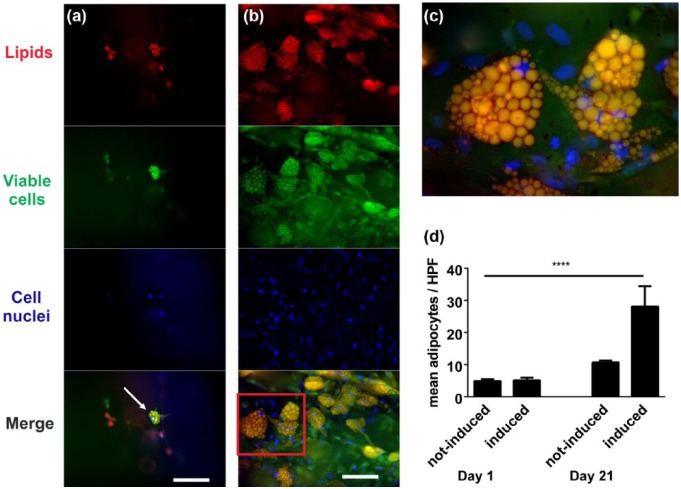
Adipogenic differentiation of hASCs in hydrogels—adipose-derived stem cells were incorporated into hydrogels and then cultured under adipogenic conditions: (a) adipoRed/fluorescein diacetate/Hoechst 33342 staining of a gelatin/hyaluronic acid composite hydrogel cultured with ADM for 7 days. Intracellular lipids = red, viable cells = green, cell nuclei = blue. In the merged image, viable cells with accumulated intracellular lipids are shown in yellow. Accumulation of intracellular lipids in viable cells could be observed in the hydrogels (arrow). Scale: 100 µm. (b) Cultured for 28 days in ADM. Scale: 100 µm, (c) merge with higher magnification (red box in (b)) of a fat-accumulating adipose-derived stem cells, and (d) mature adipocytes/HPF in adipogenic or standard medium after 21 days.

Differentiation of ASCs into mature adipocytes was induced with ADM. ASCs encapsulated in hydrogel were cultured with ADM for up to 28 days and analyzed after days 1, 7, 14, 21, and 28. After 7 days of differentiation, a few cells showed lipid accumulation (Figure 5(a)) in AdipoRed staining, and this increased significantly by day 21 (Figure 5(b) and (c)). Cells did not exhibit different phenotypes in the different hydrogel mixtures (data not shown).

### In vitro tube formation

**Figure 6.**
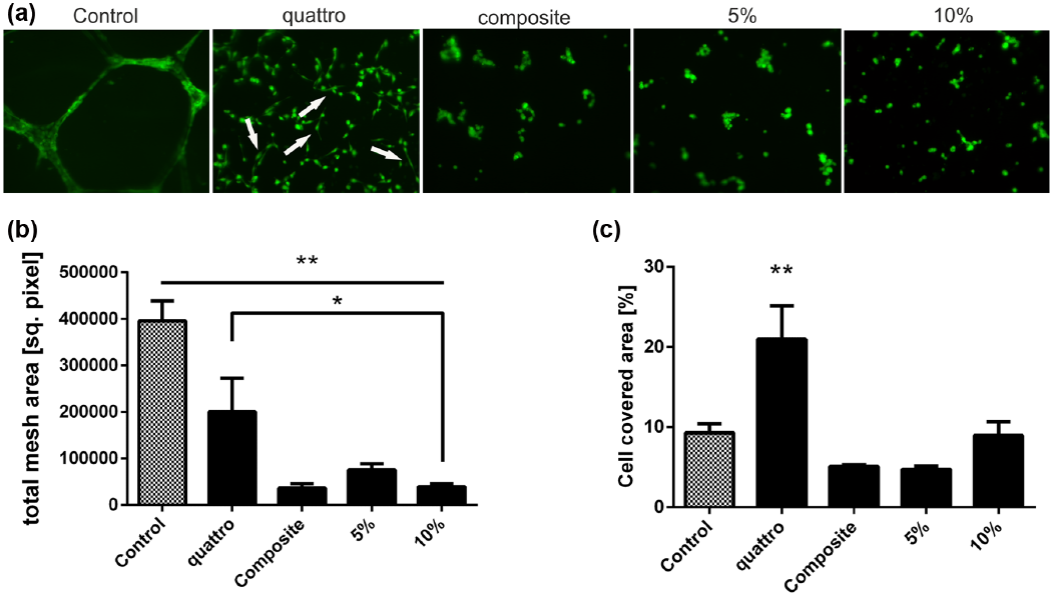
In vitro tube formation—HUVEC were seeded on hydrogels, and tube formation was analyzed after 20 h by staining with Calcein AM: (a) cells on quattroGel show changed morphology and branches (arrows), (b) analysis of mesh area showed increased tube formation on control (*p* < 0.01) compared to all groups and quattroGel compared to 10% gelatin hydrogel (*p* < 0.05), and (c) increased amount of cells were detected on quattroGel compared to all groups (*p* < 0.01).

An in vitro angiogenesis assay was performed for analysis of endothelial cells—scaffold interactions and for exploring if the hydrogels could be beneficially tailored for other cell types. HUVECs were seeded on our scaffolds and cultured with EGM-2. Matrigel was used as a positive control. After 24 h on Matrigel, HUVECs exposed a tubular network, which could not be found on the other hydrogel scaffolds (Figure 6(a)). Analysis of the network mesh area (Figure 6(b)) showed a significantly larger mesh area on Matrigel when compared with all other groups (*p* < 0.01), and on a composite hydrogel with fibrinogen and thrombin (quattroGel) when compared with the 10% methacrylated gelatin gel (*p* < 0.05). Despite no tube formation on our original scaffolds, the addition of fibrinogen and thrombin did enhance HUVEC proliferation (Figure 6(c)) and morphology (Figure 6(a)) when compared with the other hydrogels (*p* < 0.05).

## Discussion

Tissue engineering has been a rising field in the past decades and advances in this area as well as material sciences led to the emergence of the first functional organs and tissues in the recent years. Despite these achievements, a functional adipose tissue has not been successfully engineered so far. Successful tissue engineering requires precise cellular composition of the target tissue as well as vascularization. Given the wide range of synthetic or naturally derived materials available—and their possible combinations—there might be more than one way to achieve successful adipose tissue engineering.^1^ We believe that nature-derived materials, which contain proteins necessary for angiogenesis and adipogenesis, may be advantageous to this application. We attempted to create an engineered adipose tissue construct by incorporating human ASCs in a composite scaffold of methacrylated gelatin (GelMA) and HyaMA. These cells were then differentiated into an adipogenic lineage. GelMA and HyaMA^21,28,31,32,40–43^ are commonly studied in tissue engineering, and similar products are already in clinical use (e.g. HA fillers), which is convenient for further research. But despite that several things have to be taken into account.

### Scaffold characterization

The right scaffold should mimic the target tissues’ biological and biomechanical properties to ensure cell vitality, proliferation, differentiation, and matrix protein production. This should be the ultimate goal when designing a scaffold. The methacrylation of the two components allowed us to photocrosslink the components under controlled conditions. This is greatly beneficial to cell survival within gels because cross-linking can be performed with constant temperature, pH, and so on, and there is only short exposition to near UV light (365 nm).^21^ Hydrogels fabricated in this manner have good mechanical stability and swelling characteristics depending on their concentration;^32^ the latter property is important for the nutrient supply of the cells.^44,45^ Cross-linking with LAP in our study allowed us to fabricate hydrogels that were stable for at least 4 weeks. By adding cells to the soluble gel components before cross-linking, constructs can be created in various shapes depending on the used mold.

The stiffness of a scaffold has been shown to stimulate adipogenesis,^46^ and there is strong evidence that longer curing times lead to stiffer hydrogels.^47^ Previous studies showed similar storage moduli of GelMA hydrogels with mature adipocytes compared to native tissue when measured with 0.05 N.^21^ Interestingly, LAP as a photoinitiator did not exert any cytotoxic effect on the cells at the employed concentration, but on the contrary, increased cell viability and thus proved to be superior to Eosin Y in this setting. This might be due to shorter curing times (5 vs 20 min) for LAP and to reported toxicity of Eosin Y. The size of constructs is limited by the constraints of diffusion of nutrients through the scaffold. Diffusion through the scaffold requires absorption as well as transport. Water uptake or swelling of a hydrogel can indicate their capability of nutrient absorption and cell infiltration into the gel.^48^ Our scaffolds showed high swelling capacity, which was greatly enhanced in composites containing hyaluronan. This is reasonable due to the high water-binding capacity of hyaluronan.^49^ Equilibrium was reached in ~20 min in all groups, in concert with what was previously described in other macroporous hydrogels by Chang et al.^35^ and Jain et al.^50^

Our scaffolds granted a continuous supply of nutrients to the cultivated cells as supported by our data showing increased viability in the composite hydrogels when compared with gelatin hydrogels (Figure 2). Furthermore, our scaffolds mimicked the in situ composition of the ECM by comprising a combination of proteins and a glycosaminoglycan (GAG), like HA, which is the most prominent GAG in skin/soft tissue. This biomimetic condition may enhance cellular viability and ensure proliferation and differentiation. Our data support this statement. Nonetheless, hyaluronan does degrade relatively fast (data not shown), which is why these benefits are only present for the first 7–14 days of cultivation. Finally, the degradation of hyaluronan within a gelatin scaffold of higher stability may lead to larger pore size which often has been proposed to be beneficial for cell growth and differentiation.^51^ Therefore, a photocrosslinkable hydrogel made of naturally derived components such as gelatin and hyaluronan is a promising option for adipose tissue engineering.

### Cells and differentiation

Various cell types such as murine cell-line 3T3-L1, mature adipocytes,^21^ ASCs, or largely unpurified cells derived from stromal vascular fraction (SVF) have been analyzed in a 3D setting for adipose or soft tissue engineering. All these cell types have shown advantages and disadvantages in terms of cultivation, long-term survival, or comparability in previous studies. ASCs were discovered recently and are not yet accurately described. Nonetheless, they became major players in adipose tissue engineering because of their plasticity and accessability. The differences in yield and adipogenic potential of these cells depend on many factors such as donor age, type, or location of fat tissue and could affect the engineered construct and especially the cell differentiation potential.^19,52,53^ Therefore, these aspects should be considered to ensure comparable and reproducible results. Additionally, the standard isolation protocol^54^ leads to a heterogeneous cell population called SVF, which contains fibroblasts, endothelial cells, perivascular cells, macrophages, and ASCs. This fraction is often not further characterized, but rather it is cultured, and after proof of differentiation, for example, lipid accumulation, renamed to ASCs. Recent studies used the SVF to engineer a vascularized adipose tissue, which led to promising results.^22,55,56^ Therefore, we believe that it may not be necessary to further purify this fraction, and we suggest using it directly for a self-assembling approach. ASCs have become a keystone in adipose tissue engineering because they are easily accessible and are able to differentiate into various cell types including adipocytes.^19,54^

### Adipogenesis

Various scaffold materials have been shown to improve adipogenesis in vitro including gelatin,^1^ which makes it especially promising for adipose tissue engineering.

Even though ASCs differentiate well in a 2D environment under adipogenic conditions, the results in 3D culture depend on many factors.^57^ Our results showed slight differences in gene expression of cells cultivated in plastic with respect to those cultivated in GelMA or GelMA/HyaMA.

More specifically, we analyzed expression of the most important genes during adipogenesis of ASCs grown in GelMA and in GelMA/HyaMA and compared it to 2D cell culturing (TCP). These genes include Delta-like 1 (DLK1, also known as preadipocyte factor 1 (PREF-1)), peroxisome proliferator–activated receptor gamma (PPAR-γ), members of CCAAT/enhancer-binding proteins (CEBPs), fatty acid binding protein 4 (FABP4), apolipoprotein E (APOE), and ADIPOQ. DLK1 is found in ASCs but is absent in adipocytes. Therefore, DLK1 is used as a marker for preadipocytes. Consequently, down regulation of DLK1 during differentiation coincides with increased C/EBPβ and C/EBPd, which occurs prior to C/EBPα and PPAR-γ induction. CCAAT/enhancer-binding protein β (C/EBPβ) and C/EBPd are induced early during differentiation and activate PPAR-γ and C/EBPα. PPAR-γ, known to be a master regulator of adipogenesis, is necessary and sufficient to promote adipocyte differentiation. Mature adipocytes show expression of FABP4, APOE, and ADIPOQ.

Our results indicated that preadipocytes differentiated on 2D cell culture expressed some of these genes at levels even greater than those of fully differentiated preadipocytes cultivated in hydrogel components (Figure 6). Interestingly, GelMA only slightly altered expression of genes associated with fat metabolism, whereas GelMA/HyaMA mixtures induced greater alterations of gene expression compared with TCP. Nevertheless, gene expression analysis of preadipocytes grown in GelMA/HyaMA revealed no significant differences affecting the differentiation process; all the genes that we considered important were expressed, and we did not observe any impact on phenotypic differentiation. Based on these data, we suggest that both hydrogels could be well used for the establishment of in vitro fatty tissue.

### In vitro vascularization

Despite the importance of the previously discussed parameters, the key for a successful tissue-engineered construct is sufficient vascularization, which still poses a major roadblock in tissue engineering.

In vivo vascularization and in vitro vascularization are opposing approaches, and optimally should be synergistic. Most attempts for fully in vitro vascularized constructs remained unsuccessful so far. Nonetheless, the growth of endothelial cells and their formation can indicate the feasibility of scaffolds for in vitro vascularization. We used a common in vitro tube formation assay to analyze the growth of HUVECs. The cells remained vital on all scaffolds; however, the GelMA and GelMA/HyaMa composites did not promote tube formation. However, addition of fibrinogen and thrombin (quattroGel) enhanced endothelial proliferation and vascular morphogenesis, especially in presence of vascular endothelial growth factor (VEGF).^58,59^ Co-cultures of ASCs and endothelial cells in quattroGel may show good results because ASCs secrete angiogenetic factors,^60–62^ which could increase in vitro vascularization. Thus, these hydrogels can work as building blocks in adipose tissue engineering, which can be altered with a variety of proteins to address the needs of different approaches.

## Conclusion

We could produce stable, yet cytocompatible, 3D scaffolds with methacrylated gelatin and hyaluronan, which granted viability and differentiation to encapsulated ASCs for longer periods. This stability allowed the cells to survive over a period of 3–4 weeks and enabled the targeted differentiation into adipogenic lineage within the matrix as well. Combined GelMA/HyaMA hydrogels show an advantage in adipogenic differentiation and viability compared to GelMA hydrogels. With additional ECM/protein components, these scaffolds could be tailored sufficiently for soft tissue engineering. Nonetheless is a sufficient vascularization still problematic for these hydrogels and needs further research especially in vivo studies using appropriate models.

## Supplementary Material

Supplementary material
