# Supplementary material for: Methacrylated gelatin/hyaluronan-based hydrogels for soft tissue engineering
Source: J Tissue Eng. 2017 Dec 21;8:2041731417744157. doi: 10.1177/2041731417744157 (PMC5753891; doi:10.1177/2041731417744157)
Supplement: Supplementary material [file Supplementary_File_-_Table_of_genes.pdf]

Gene name	Description
18S	18S ribosomal RNA
ABHD5	abhydrolase domain containing 5
ACLY	ATP citrate lyase
ACSL4	acyl-CoA synthetase long chain family member 4
ADIPOQ	adiponectin, C1Q and collagen domain containing
ADRA2A	adrenoceptor alpha 2A
ADRA2B	adrenoceptor alpha 2B
ADRA2C	adrenoceptor alpha 2C
ADRB1	adrenoceptor beta 1
ADRB2	adrenoceptor beta 2
AGT	angiotensinogen
APOE	apolipoprotein E
CAV1	caveolin 1
CEBPA	CCAAT/enhancer binding protein alpha
CEBPB	CCAAT/enhancer binding protein beta
CEBPD	CCAAT/enhancer binding protein delta
DGAT1	diacylglycerol O-acyltransferase 1
DLK1	delta like non-canonical Notch ligand 1
FABP4	fatty acid binding protein 4
FASN	fatty acid synthase
GAPDH	glyceraldehyde-3-phosphate dehydrogenase
GK	glycerol kinase
GPD1	glycerol-3-phosphate dehydrogenase 1
IGF1R	insulin like growth factor 1 receptor
IL1B	interleukin 1 beta
IL6	interleukin 6
IRS1	insulin receptor substrate 1
LEP	leptin
LIPE	lipase E, hormone sensitive type
LPL	lipoprotein lipase
MGLL	monoglyceride lipase
PDE3B	phosphodiesterase 3B
PKIG	cAMP-dependent protein kinase inhibitor gamma
PLIN1	perilipin 1
PNPLA2	patatin like phospholipase domain containing 2
PPARG	peroxisome proliferator activated receptor gamma
PRKAA1	protein kinase AMP-activated catalytic subunit alpha 1
PRKAA2	protein kinase AMP-activated catalytic subunit alpha 2
PRKACA	protein kinase cAMP-activated catalytic subunit alpha
PRKG1	protein kinase, cGMP-dependent, type I

PTGES2	prostaglandin E synthase 2
SIRT1	sirtuin 1
SLC27A1	solute carrier family 27 member 1
SLC27A4	solute carrier family 27 member 4
SLC2A4	solute carrier family 2 member 4
SREBF1	sterol regulatory element binding transcription factor 1
TNF	tumor necrosis factor
VEGFA	vascular endothelial growth factor A
